# Supplementary material for: Integrating Hi-C links with assembly graphs for chromosome-scale assembly
Source: PLoS Comput Biol. 2019 Aug 21;15(8):e1007273. doi: 10.1371/journal.pcbi.1007273 (PMC6719893; doi:10.1371/journal.pcbi.1007273)
Supplement: S1 Text — (DOCX) [file pcbi.1007273.s001.docx]

## Best Buddy Scoring Effectiveness

For correct scaffolding, we want to filter false edges and retain only the correct linkage information between pairs of unitigs. Our previous algorithm used a fixed, user-defined minimum for edges connecting a pair of unitigs. The drawback of a fixed cutoff is that it cannot handle variations in coverage within the assembly and varies between any pair of sequencing datasets. To compare the scoring methods, we down-sample the alignments into three different sets with 0.25, 0.5 and 0.75 of the original coverage and computed the precision of filtering based on the ratio score and a fixed threshold. The precision remained almost constant for the ratio cutoff on all datasets, whereas the precision changes rapidly for different coverages and a fixed threshold (Supplementary Figure S1).

## Commands used for analysis

NA12878 + Arima-HiC scaffolding

SALSA2 with graph:

python run_pipeline.py -a asm.unitigs.fasta -l asm.unitigs.fasta.fai -b v3_alignment.bed -o scaffolds_with_graph -c 10000 -e GATC,GANTC -g asm.unitigs.gfa -m yes

SALSA2 without graph:

python run_pipeline.py -a asm.unitigs.fasta -l asm.unitigs.fasta.fai -b v3_alignment.bed -o scaffolds_with_graph -c 10000 -e GATC,GANTC -m yes

3D-DNA with breaking:

sh 3d-dna/run-pipeline.sh -m haploid -t 10000 -s 2 asm.fasta merged_nodups.txt

NA12878 + Mitotic HiC scaffolding

SALSA2 with graph:

python run_pipeline.py -a asm.unitigs.fasta -l asm.unitigs.fasta.fai -b v3_alignment.bed -o scaffolds_with_graph -c 10000 -e AAGCTT

-g asm.unitigs.gfa -m yes

3D-DNA with breaking:

sh 3d-dna/run-pipeline.sh -m haploid -t 10000 -s 2 asm.fasta merged_nodups.txt

3D-DNA without breaking:

sh 3d-dna/run-pipeline.sh -m haploid -t 10000 -s 0 asm.fasta merged_nodups.txt

NA12878 + Chicago scaffolding:

SALSA2 with graph:

python run_pipeline.py -a asm.unitigs.fasta -l asm.unitigs.fasta.fai -b v3_alignment.bed -o scaffolds_with_graph -c 10000 -e GATC

-g asm.unitigs.gfa -m yes

3D-DNA with breaking:

sh 3d-dna/run-pipeline.sh -m haploid -t 10000 -s 2 asm.fasta merged_nodups.txt

3D-DNA without breaking:

sh 3d-dna/run-pipeline.sh -m haploid -t 10000 -s 0 asm.fasta merged_nodups.txt
