# Supplementary figures and images for: Integrating Hi-C links with assembly graphs for chromosome-scale assembly
Source: PLoS Comput Biol. 2019 Aug 21;15(8):e1007273. doi: 10.1371/journal.pcbi.1007273 (PMC6719893; doi:10.1371/journal.pcbi.1007273)

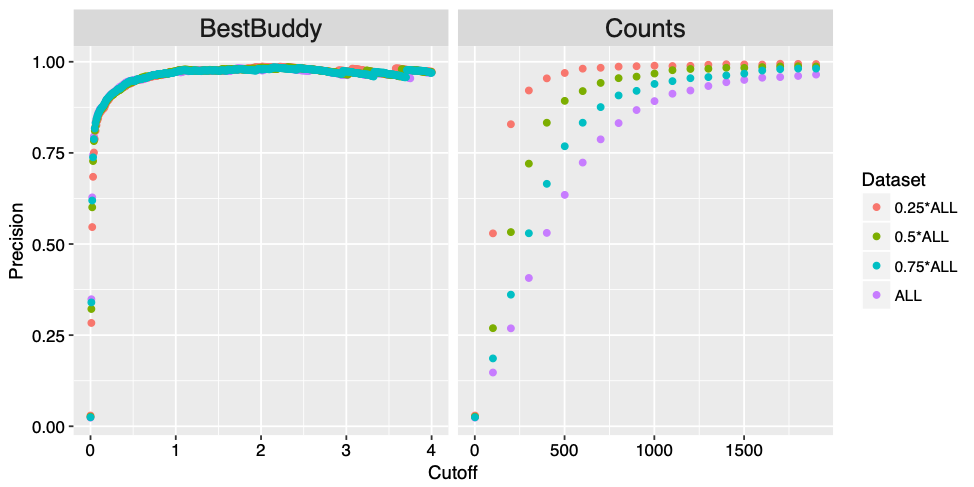

Supplement: S1 Fig — The plot on the left shows the curve for the SALSA2 best buddy weight cutoffs and the plot on the right shows the curve for a fixed Hi-C pair count cutoff, used in SALSA1, across changing coverage. (PNG) [file pcbi.1007273.s002.png]

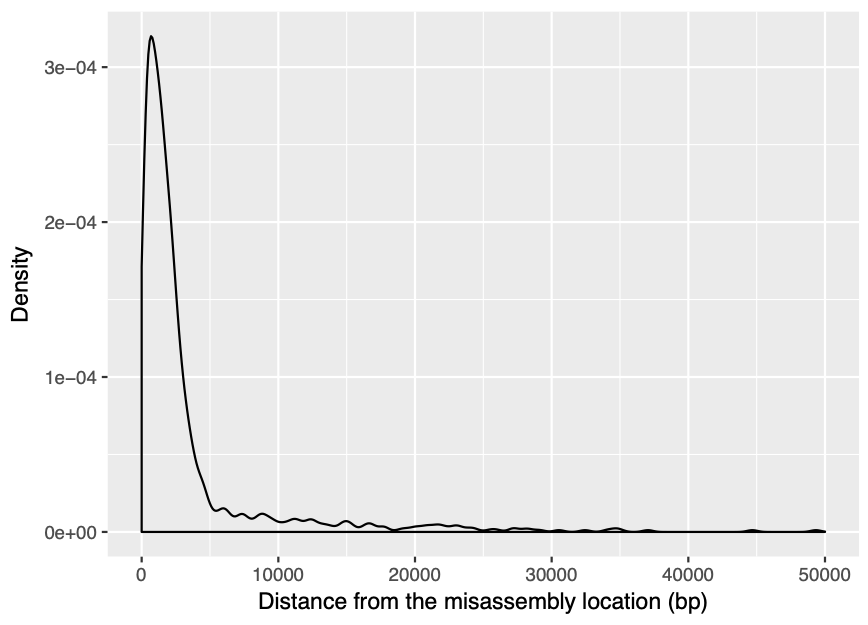

Supplement: S2 Fig — (PNG) [file pcbi.1007273.s003.png]
