# Supplementary material for: Integrating Hi-C links with assembly graphs for chromosome-scale assembly
Source: PLoS Comput Biol. 2019 Aug 21;15(8):e1007273. doi: 10.1371/journal.pcbi.1007273 (PMC6719893; doi:10.1371/journal.pcbi.1007273)
Supplement: S1 Table — (DOCX) [file pcbi.1007273.s004.docx]

| **Unitig Size** | **Sensitivity** | **Specificity** |
| --- | --- | --- |
| 100 kbp | 31% | 78.5% |
| 200 kbp | 50% | 81% |
| 300 kbp | 68.5% | 80% |
| 400 kbp | 77.5% | 82.5% |
| 500 kbp | 94% | 85.5% |
| 600 kbp | 94.5% | 88.5% |
| 700 kbp | 92.5% | 89% |
| 800 kbp | 95% | 89.5% |
| 900 kbp | 90% | 91% |
