# Supplementary material for: Integrating Hi-C links with assembly graphs for chromosome-scale assembly
Source: PLoS Comput Biol. 2019 Aug 21;15(8):e1007273. doi: 10.1371/journal.pcbi.1007273 (PMC6719893; doi:10.1371/journal.pcbi.1007273)
Supplement: S2 Table — (DOCX) [file pcbi.1007273.s005.docx]

| **Unitig Size** | **Sensitivity** | **Specificity** |
| --- | --- | --- |
| 100 kbp | 38.5% | 100% |
| 200 kbp | 44% | 100% |
| 300 kbp | 58.5% | 100% |
| 400 kbp | 61% | 100% |
| 500 kbp | 62.5% | 100% |
| 600 kbp | 77.5% | 100% |
| 700 kbp | 85.5% | 100% |
| 800 kbp | 89% | 100% |
| 900 kbp | 92.5% | 100% |
