# Supplementary material for: Integrating Hi-C links with assembly graphs for chromosome-scale assembly
Source: PLoS Comput Biol. 2019 Aug 21;15(8):e1007273. doi: 10.1371/journal.pcbi.1007273 (PMC6719893; doi:10.1371/journal.pcbi.1007273)
Supplement: S3 Table — The input assembly had contig N50 of 631.72 kbp. (DOCX) [file pcbi.1007273.s006.docx]

| **Method** | **#Scaffolds** | **Max Scaffold Size (Mbp)** | **N50 (Mbp)** | **NA50 (Mbp)** | **Orientation Errors** | **Ordering Errors** | **Chimeric Errors** |
| --- | --- | --- | --- | --- | --- | --- | --- |
| **SALSA2** | 282 | 48.016 | 8.15 | 8.04 | 19 | 21 | 231 |
| **3D-DNA** | 619 | 104.61 | 46.36 | 11.75 | 28 | 21 | 290 |
