# Supplementary material for: Integrating Hi-C links with assembly graphs for chromosome-scale assembly
Source: PLoS Comput Biol. 2019 Aug 21;15(8):e1007273. doi: 10.1371/journal.pcbi.1007273 (PMC6719893; doi:10.1371/journal.pcbi.1007273)
Supplement: S4 Table — The input assembly had contig N50 of 94.25 kbp. We could not compute errors for duplicated assembly as reference genome is primary only and aligning duplicated contigs to haploid reference makes it hard to define true orientation and ordering. (DOCX) [file pcbi.1007273.s007.docx]

| **Method** | **#Scaffolds** | **Max Scaffold Size (Mbp)** | **N50 (kbp)** | **NA50 (Mbp)** | **Orientation Errors** | **Ordering Errors** | **Chimeric Errors** |
| --- | --- | --- | --- | --- | --- | --- | --- |
| **SALSA2** | 5074 | 9.25 | 840.12 | NA | NA | NA | NA |
| **3D-DNA** | 11939 | 8.51 | 72.69 | NA | NA | NA | NA |
